# OXTR Gene DNA Methylation Levels Are Associated with Discounting Behavior with Untrustworthy Proposers

**DOI:** 10.3390/brainsci12010098

**Published:** 2022-01-12

**Authors:** Stefano Anzani, Loreta Cannito, Fabio Bellia, Alberto Di Domenico, Bernardo Dell’Osso, Riccardo Palumbo, Claudio D’Addario

**Affiliations:** 1 Department of Neuroscience, Imaging and Clinical Sciences, University “G. d’ Annunzio” of Chieti-Pescara, 66100 Chieti, Italy; s.anzani92@gmail.com; 2 Department of Psychological, Health and Territorial Sciences, University “G. d’ Annunzio” of Chieti-Pescara, 66100 Chieti, Italy; loreta.cannito@unich.it (L.C.); alberto.didomenico@unich.it (A.D.D.); 3 Center for Advanced Studies and Technology, University “G. d’ Annunzio” of Chieti-Pescara, 66100 Chieti, Italy; 4 Faculty of Bioscience and Technology for Food, Agriculture and Environment, University of Teramo, 64100 Teramo, Italy; fbellia@unite.it; 5 Department of Psychiatry, Department of Biomedical and Clinical Sciences “Luigi Sacco”, University of Milan, ASST Fatebenefratelli-Sacco, 20019 Milan, Italy; bernardo.dellosso@unimi.it; 6 Department of Clinical Neuroscience, Karolinska Institute, 10316 Stockholm, Sweden

**Keywords:** temporal discounting, probability discounting, trust, oxytocin, DNA methylation, oxytocin receptor

## Abstract

Individual differences in temporal and probabilistic discounting are associated with a wide range of life outcomes in literature. Traditional approaches have focused on impulsiveness and cognitive control skills, on goal-oriented personality traits as well as on the psychological perception of time. More recently, literature started to consider the role of social and contextual factors in discounting behavior. Between others, higher generalized trust in human beings and specific trust in people who will deliver the future/probabilistic rewards have been related to a stronger willingness to wait and to assume risk. Moreover, the tendency to trust others has been associated with the oxytocin receptor gene regulation that can be modified by life experiences. In this perspective, we hypothesized that differences in the tendency to wait and to take risks for a more desirable reward according to the proposer’s trustworthiness could be related to a different level of DNA methylation at the oxytocin receptor gene. Findings confirmed that participants are less willing to wait and to risk when the proposer is considered highly untrustworthy and revealed how higher oxytocin receptor gene DNA methylation is associated with a stronger effect due to the presence of an untrustworthy proposer. Limits and future directions are outlined.

## 1. Introduction

Most of the decision-making problems we face everyday concern choices whose consequences will be clear over time. This is the case, for example, with choices concerning investments, savings, and consumption, with choices related to one’s lifestyle or health, as well as many problems of political economy. More specifically, intertemporal choices and risky choices are considered relevant in determining the level of well-being that an individual can enjoy [1,2].

Discount behaviors (both temporal and risk) have been widely investigated and considered generally stable within the individual [3]. They are linked to the development of numerous sub-optimal and pathological behaviors [4], related to physical exercise in adults [5] and the elderly [6], to the use of alcohol and drugs [7], to the involvement in shopping or gambling addictions or in savings behavior [8,9,10], as well as to sexual and food diseases [11,12].

Among others, individual differences in willingness to delay a gratification or to prefer a sure option instead of a risky one has traditionally been related to cognitive control skills, to impulsiveness, to the psychological perception of time, and to personality traits such as goal orientation and sensation seeking [13,14].

Just recently those traditional accounts have been accompanied by other possible explications related to more social and contextual factors.

For example, it has been reported that children tend to increase their willingness to wait in order to obtain a more desirable reward when the delivering person is considered trustworthy [15,16].

It has been also shown that trust plays a role in delaying gratification even when children have no information about the individual who is promising the future reward (neither face nor behavior) since just a higher level of generalized trust in humans was enough to wait longer [17].

Trust seems to be crucial for many processes in our everyday life, contributing to social and personal success. It is indispensable in friendship, love, families, and work organizations and plays a key role in economic exchanges and politics [18].

Our ability to trust others can be considered multifactorial since it depends upon our previous life experiences, but it is also biologically determined. From a biological point of view, several studies suggest a role for different genes in trust including those belonging to serotonin, arginine-vasopressin, and oxytocin systems [19], with the latter in particular the recent object of several studies. Oxytocin is one of the brain’s most abundant neuropeptides, involved in several physiological responses including social behaviors [20]. To elicit its effect, oxytocin must bind its receptor (OXTR) which is widely distributed in the brain, including the middle insula and in the anterior cingulate cortex, which are known to be part of the so-called social brain [21]. The production and release of oxytocin and its activity on OXTR in specific brain regions affect our tendency to feel trust in others, which can be considered one of the most crucial social emotions. This is confirmed from several studies measuring endogenous levels of oxytocin and applying the intranasal inhalation of oxytocin. The first evidence of a relationship with social behaviors was that receiving a signal of trust was associated with higher levels of peripheral oxytocin, and that this endogenous level was also related to trustworthy behavior [22,23]. This was then further reinforced showing how during a classic trust game participants who received the exogenous oxytocin were more willing to trust an (until then unknown) investor and to take more financial risks, compared to the control group [24]. Moreover, the oxytocin system has also been related to some disorders’ pathophysiology, such as autism, for which a therapeutic use of this peptide hormone has been suggested to promote social skills [25]. More recently, the role of oxytocin signaling in the amygdala in different social-based processes was highlighted [26,27].

It seems, therefore, that the oxytocin system plays a key role in the formation of social bonds, attachment, and the memory social system. One approach to study the role of the oxytocin system in relational and social skills concerns the influence of OXTR gene polymorphisms in promoting a secure attachment style [28] in predicting risky decision-making [29] affecting social anxiety symptom development [30] as well as influencing social behavior in general [31].

However, besides the role of genetics in trust, recent evidence suggests the relevance of the interactions between gene and environment that can evoke alterations in genes’ expression through epigenetic mechanisms [32]. Thus, for example, chronic experiences of stress (e.g., low socioeconomic status—SES) or trauma (e.g., abuse during childhood) throughout life can induce immune dysfunctions as consequence of epigenetic modifications, and so doing, produce a change in attitudes and behaviors [33]. For instance, low SES modifies the extent to which people can expect to realize deferred rewards, leading to more present-oriented behavior in a range of domains [34].

The most investigated epigenetic mechanism is DNA methylation involving the transfer of a methyl group to the C-5 position of the cytosine (C) pyrimidine ring when this nucleotide is followed in the linear sequence, along the 5′ → 3′ direction, by a guanine residue (CpG site). This modification is involved in transcriptional silencing [35].

Of relevance, it has been reported that a reduction in the OXTR gene DNA methylation, leading to higher OXTR expression, in subjects showing more secure attachment styles, improved the ability to recognize emotional facial expressions, greater superior temporal sulcus activity during social-cognitive tasks, and larger fusiform gyrus grey matter volume suggesting a general higher competence in social skills [36]. Moreover, a preclinical study showed that early (negative) experiences can regulate OXTR DNA methylation in the nucleus accumbens [37].

Given this evidence, our aim is to verify if the presence of an untrustworthy proposer changes participants’ discounting behavior by decreasing both their willingness to wait for and to take risk for a larger reward (i.e., increased preference for smaller immediate over larger delayed, and preference for smaller sure over larger not sure rewards). To this end, we developed two discounting tasks (described in detail in the next section) where we manipulated the presence of proposers at various levels of trustworthiness by showing an image of their face, a method that has been used to elicit an emotional reaction in participants [38,39]. Moreover, we attempt to analyze and correlate behavioral outcomes with participants’ OXTR gene DNA methylation levels.

## 2. Materials and Methods

### 2.1. Sample

Sixty right-handed neurotypical volunteers (26 males, 34 females. Mean age: 24.2 ± 2.9 SD) with no psychiatric or drug addiction history participated in the study after providing written informed consent in accordance with the ethical standards of the 1964 Declaration of Helsinki. The research protocol was approved by the Institutional Review Board of Psychology (IRBP) of the Department of Psychological, Health and Territorial Sciences at G. d Annunzio University of Chieti-Pescara (identification code: 20026; date of approval: 19 February 2021). Participants were undergraduate and graduate students from the same University. Compensation, in the form of monetary payment, was provided for all participants by pulling out one random selected choice from one of the two decision tasks they completed, so the range of possible earnings was between 11€ and 100€. This was done to ensure that their choices reflected their preference for that trial.

Participants individually arrived at the laboratory and received a form containing information about procedures. The whole experimental session was conducted in a quiet and private environment by the same researcher.

### 2.2. Salivary Samples Collection

As the first activity, participants’ salivary samples were collected by using a standard kit (Salivette, Sarstedt, Numbrecht, Germany). Saliva was chosen since it has technical advantages over blood, particularly that it is a non-invasive sampling method, and several molecular measures in saliva might reflect those in blood [40]. To ensure a correct sampling, during the recruiting phase participants were asked not to take food, drugs, drinks (besides water), or use lip products as well as not to smoke or brush their teeth at least two hours before arriving at the laboratory to avoid possible contamination. Each sample was then stored at −20 °C before being analyzed.

### 2.3. Discounting Tasks

After salivary sampling, participants completed two different tasks whose presentation order was counterbalanced between participants.

In one task, participants’ temporal discounting behavior was assessed by using the extensively validated and commonly used 27-item MCQ—Monetary Choice Questionnaire [41]. In the MCQ, on each item, participants chose between immediate, smaller rewards (e.g., €25 today) and delayed, larger rewards (e.g., €35 in 25 days) of three differing magnitudes (9 small rewards, 9 medium, and 9 large).

**Figure 1:**
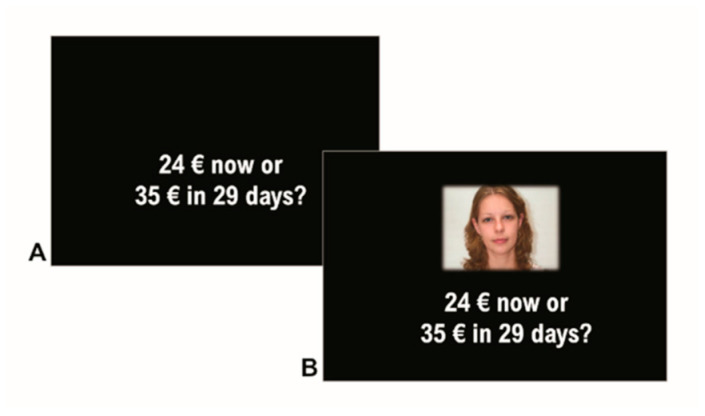
Behavioral Paradigm. Participants were asked to press the “A” key if they preferred the immediate reward in the MCQ and sure reward in the PDQ and to press the “L” key if they preferred the delayed reward in the MCQ and the probabilistic reward in the PDQ. Choice items were randomly presented in the baseline condition (proposer face was not shown) and in six face conditions as obtained by manipulating face gender (male, female) and face trustworthiness (trustworthy, neutral, untrustworthy). (**A**) Schematic representation of a trial in the baseline condition for the MCQ. (**B**) Schematic representation of the same trial in the “face” condition, with the picture of a proposer shown with the MCQ item.

Each item was presented 7 times. In the “baseline” block (Figure 1A), participants were just presented the two alternatives and asked to choose, as soon as possible, the more appealing one. In the “face” blocks (Figure 1B), in addition to the alternatives, participants were presented one of six different faces. They were then instructed to imagine that the person shown in the picture was the one making the proposal currently on the screen. As a result, participants answered a total of 189 items presented in randomized order.

The six different Caucasian faces were previously selected from a face database [42] after asking an independent sample to rate each face for trustworthiness by using a 7-point Likert scale (from 1 = completely untrustworthy to 7 = completely trustworthy). This allowed us to pick out 3 male face pictures (untrustworthy, neutral, and trustworthy) and 3 female face pictures (untrustworthy, neutral, and trustworthy) to be used as proposers in the “face” conditions.

In the other task, participants’ probabilistic discounting behavior was assessed by using the 30-item PDQ—Probability Discounting Questionnaire [43]. In the PDQ, on each item, participants chose between a sure, smaller reward (e.g., €20 for sure) and a larger amount of money delivered probabilistically (e.g., 10% of winning €80). The probabilistic discount indeed was used to investigate the effect of reward probability on decision-making by determining the amount to be received for sure that is equally preferred to a risky outcome. The questionnaire is composed of three blocks of 10 items each one comparing different rewards at different probabilities (Block 1: €20 vs. €80, Block 2: €40 vs. €100, Block 3: €40 vs. €60).

As for the MCQ, participants were randomly presented each one of the 30 items in the “baseline” condition (no proposer face was shown) and in six “face” conditions (untrustworthy male, untrustworthy female, neutral male, neutral female, trustworthy male, and trustworthy female proposer).

Both tasks were presented via computer using a 15.5″ LCD monitor (1366 × 768 pixels). The software package E-Prime 3.0 was used for stimulus presentation and response sampling. Participants, seated in front of the monitor at about 60 cm, were instructed on how to use the keyboard to answer. Half of the participants were instructed to press “A” key if they preferred the “Now” option (for the MCQ task) and the “Sure” option (for the PDQ task), and to press the “L” key if they preferred the “Later” option (for the MCQ task) and the “Not sure” option (for the PDQ task). To ensure that results were not affected by key side (A on the left and K on the right), the other half of the participants completed the two tasks after being instructed to use the two keys in the opposite sense (“A” for “Later” and “Not Sure”; “L” for “Now” and “Sure”).

### 2.4. DNA Methylation Study

Genomic DNA from buccal swab samples (Salivette, Sarstedt, Numbrecht, Germany) was prepared using the salting-out method as described previously [44]. The NanoDrop 2000c UV-Vis Spectrophotometer (Thermo Fischer Scientific, Waltham, MA, USA) was used to assess the quantity and quality of each sample. The ratio of optical density at 260 and 280 nm was used to assess protein contamination: a value of 1.8 was considered acceptable. Each purified DNA was subjected to bisulfite modification by means of the EZ DNA Methylation-GoldTM Kit (Zymo Research, Orange, CA, USA), according to the manufacturer’s protocol. The DNA methylation status of each of the CpG sites in *OXTR* CpG island located in exon III was assessed using a pyrosequencing assay. Bisulfite treated DNA was first amplified by the PyroMark PCR Kit (Qiagen, Hilden, Germany) with a biotin labelled primer (Hs_OXTR_01_PM PyroMark CpG assay, PM00016821) according to the manufacturer’s recommendations. PCR conditions were as follows: 95 °C for 15 min, followed by 45 cycles of 94 °C for 30 s, 56 °C for 30 s, 72 °C for 30 s, and, finally, 72 °C for 10 min.

**Figure 2:**
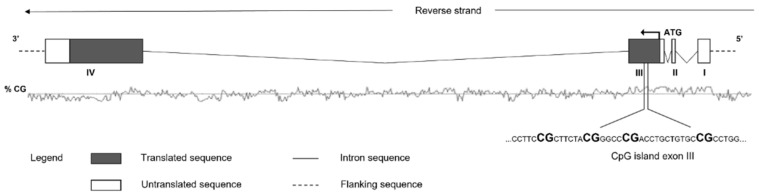
Schematic representation of human *OXTR* gene. Transcription start site position, exons, and introns are depicted, as well as region sequenced to analyze DNA methylation levels of the 4 CpG sites (in bold).

PCR products were then verified by agarose electrophoresis. The sequencing was performed on a PyroMark Q24 ID using Pyro Mark Gold reagents (Qiagen, Hilden, Germany), after immobilizing PCR products to Streptavidin Sepharose High-Performance (GE Healthcare, Chicago, IL, USA) beads via biotin affinity and denatured to allow the annealing with the sequencing primers. Within the CpG island depicted in Figure 2, we chose to analyze the percentage of DNA methylation of 4 CpG sites. The methylation’s level was analyzed through the PyroMark Q24 ID version 1.0.9 software which calculates the methylation percentage mC/(mC + C) (mC = methylated cytosine, C = unmethylated cytosine) for each CpG site, allowing quantitative comparisons.

Quantitative methylation results were expressed both as a percentage of every single CpG site and as the average of the methylation percentage of all the CpG sites investigated (see Figure 2).

### 2.5. Behavioral Analysis

Based on the participants’ observed behavior, we calculated k scores and h score by using an R syntax [45]. The syntax is based on the following well-known equations, each containing a single free parameter which is interpreted as degree of delay (k) or probability (h) discounting. When the free parameter value increases, the subjective value of the delayed or probabilistic outcome is more steeply discounted. For the MCQ, discounting rates for each level were calculated using Mazur’s [46] and Kirby and colleagues’ [41] hyperbolic discounting equation:

V = A/(1 + kD),(1)

where V is the present value of the delayed reward, A is the amount of the delayed reward, D is the delay, and k is the individual discounting rate. The discounting rate (k) represents the slope of the hyperbolic function, the individual’s value of delayed rewards, with larger k values reflecting larger delay discounting. Therefore, k describes the steepness of the discounting curve or, in other words, the degree to which a monetary value is devalued over time. For this task each one of the 27 items is classified according to the k rank. The k rank classifies items in 9 different groups and is defined based on the k indifference. The k indifference is the value of the discount rate at which the immediate and delayed rewards are of equal value according to Equation (1).

A similar procedure was applied to assess probabilistic discounting. In this case, the delay D is replaced by the odds against winning, Θ = (1 − *p*)/*p*, as reported in the Equation (2) which describes hyperbolically declining subjective values of probabilistic outcomes:

V = A/(1 + h Θ),(2)

As for the MCQ, also in the PDQ, each one of the 30 items is classified according to the h rank. The h rank reflects the degree of probability discounting at indifference between the certain and the probabilistic outcome. Thus, we obtained an individual k value—for which the higher the k value, the more steeply the individual discounts’ rewards delayed in time—and an individual h value—for which the higher the h value the more steeply the probabilistic reward is discounted.

### 2.6. Statistical Analyses

One participant was excluded from the MCQ and two participants from the PDQ analysis due to technical errors in the computer session. In total, 9 participants were not included in the final moderation analysis because of a mistake during salivary sampling. Thus, the final sample sizes for the behavioral analyses were 59 and 58 for the MCQ and PDQ, respectively, and 48 for the analysis on methylation levels. Most statistical analyses were conducted using R. We used linear mixed effects models to analyze discounting parameters from the two tasks. We chose to use multilevel models instead of classical ANOVA methods because they allow for the non-independence that we had in our data, having multiple observations from each subject, explicitly declaring the structure of the random effects accordingly. The models were coded using the *lmer* function from the *lme4* R package [47]. Since discounting parameters measured with the choice questionnaire are often skewed, we applied a log transformation as often suggested in the literature [45].

To analyze response times, first we filtered out implausibly fast and slow trials (shorter than 250 ms or longer than 3 SD over the mean computed for each subject). Again, we used mixed effects models, this time using the *glmer* function from the *lme4* that allowed us to utilize an inverse Gaussian distribution which better represents that of response times, without applying any transformation to the data [48]. We chose to log-transform the discounting parameters and not the response times because parameters on the original scale are not easier to interpret than their log-transformed counterparts, whereas we have a better sense and understanding of response times expressed in units of time compared to their log-transformed counterparts.

Some of the models include higher level interactions, which are not easy to interpret from the coefficients of the model. For this reason, we used the *Anova* function from the *car* R package to obtain ANOVA-like omnibus tests of effects through Wald chi square tests [49]. In order to obtain the proper main effects and interactions, and not simple effects, we coded the categorical predictors of these models using sum contrast coding [50].

To carry out post hoc multiple comparisons of significant effects and compute estimated marginal means, we used the R package *emmeans* with Bonferroni correction for multiple comparisons [51].

Moderation analyses were performed using the PROCESS Macro Package for SPSS [52].

## 3. Results

We will report results for the two tasks separately, and for each task we will present results on discounting parameters and response times. At the end, we will report the results of a moderation analysis with the methylation data.

### 3.1. Delay Discounting

#### 3.1.1. Discounting Parameter k

To test for the effect of the proposer on the change in the discounting parameter k, we use a linear mixed effect model, with the current proposer as the predictor of the log-transformed k value, and with a random intercept for each participant, accounting for individual differences and for the repeated measures design.

**Figure 3:**
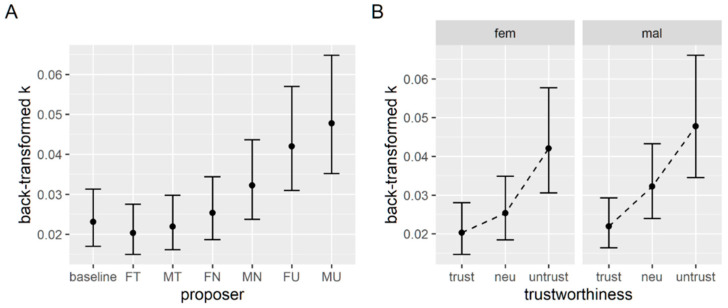
(**A**) Estimated marginal means of k parameters from the first model comparing baseline and each experimental condition. Labels on the x axis indicate the gender (female or male) and trustworthiness (trustworthy, neutral, untrustworthy). (**B**) Estimated marginal means of k parameters for each level of trustworthiness, with female (left) and male (right) proposers.

The model uses treatment coding for factors, with the baseline set as the reference value, so each coefficient of the model can be used to test the change in k due to each proposer. We apply the t as z criterion for significance of the coefficients, so t values higher than two can be considered significant. Even though this method has been shown to be anti-conservative, this mostly applies to much smaller sample sizes [53]. As reported in Table 1, both untrustworthy proposers and the male neutral proposer elicit a significant increase in discounting rate; therefore, compared to the baseline condition participants, they show less willingness to wait. The estimated marginal means of the back-transformed k values are plotted in Figure 3A.

In order to test the different contributions of the proposers’ features, we use a second mixed effect model with gender and trustworthiness of the proposer as fixed factors and a random intercept for each subject, this time excluding the data regarding the baseline condition (for which the tested factors are meaningless). In order to get proper omnibus tests of the main effects and interactions, the factors are recoded with sum contrasts coding [50].

Omnibus tests (type 3 Wald chi square tests) reveal that the effect of the trustworthiness level is significant, as well as the effect of gender, but their interaction is not (see Table 2). Post hoc comparisons with Bonferroni correction show that the discounting parameter is significantly higher when the proposer is untrustworthy (0.045 ± 0.007) compared to trustworthy (0.021 ± 0.003) and neutral (0.029 ± 0.004). Figure 3B shows the estimated marginal means for the back-transformed k parameters in each tested condition.

#### 3.1.2. Response Times

**Figure 4:**
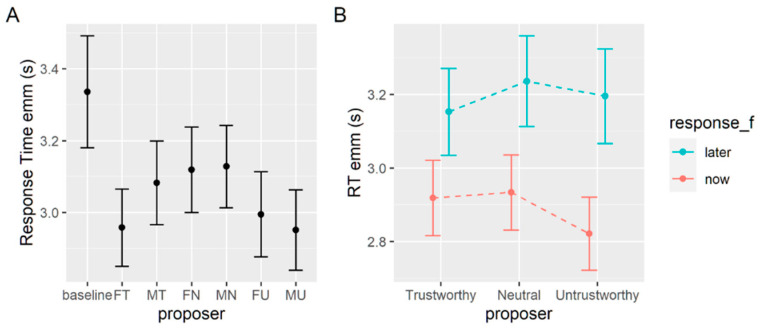
(**A**) Estimated marginal means of response times in seconds for the baseline and each experimental condition in the delay discounting task. (**B**) Estimated marginal means of response times for each level of trustworthiness when the preference was for the immediate (red) or delayed (blue) options. Only the main effect of response is significant, but the plot shows the non-significant interaction for ease of comparability with similar figures in the paper.

A second analysis is carried out on response times. On average, response times in seconds (s) in this task are 2.88 (SD = 1.84) s, 3.11 (1.68) s without a face, and 2.84 (1.62) s with a face. We use a generalized mixed effect model with family set to inverse Gaussian and identity link functions. This allows us to consider the skewed distribution that is typical of response times without the need to transform the variable [48]. We use the current proposer as the fixed effect with the baseline set as the reference level and we allow the random intercept to vary for each subject. Again, each coefficient tells us the difference between the baseline condition (with no proposer) and the other conditions. Each coefficient is significant and negative, meaning that when a proposer is present, participants take less time to decide compared to the baseline condition (see Table 3 and Figure 4A).

Again, to test for different contributions of the factor at play, we use a second model, without data from the baseline condition, with gender, trustworthiness of the proposer, and the response given by the subject as fixed effects with two and three-way interactions, and a random intercept for each subject. The response variable is the two levels factor that carries the information on which of the two alternatives the subject ends up preferring, either the immediate but smaller one or the delayed but larger one.

The omnibus Wald chi square test (see Table 4) shows that the only significant effect is given by a given response, and a post hoc comparison reveals that participants are faster to get to a decision when this decision is the immediate option (2.89 ± 0.05) rather than the delayed option (3.19 ± 0.05). No main significant effect of gender or of the proposer’s trustworthiness are detected. The estimated marginal means are shown in Figure 4B.

### 3.2. Probability Discounting

#### 3.2.1. Discounting Parameter h

**Figure 5:**
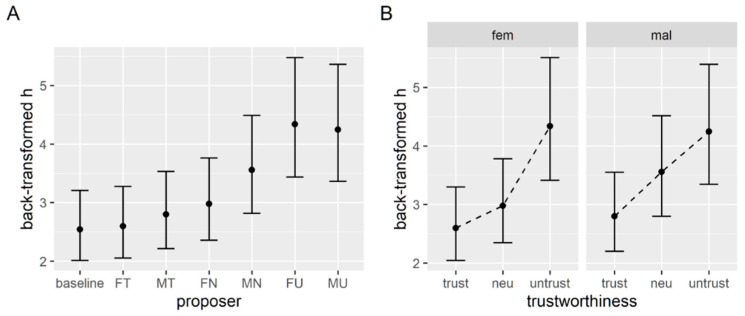
(**A**) Estimated marginal means of h parameters from the first model comparing baseline and each experimental condition. (**B**) Estimated marginal means of h parameters for each level of trustworthiness, with female (left) and male (right) proposers.

Analyses for the probabilistic task closely follow the ones described in the previous section. A mixed effect model on the log-transformed h parameter, with the current proposer as the fixed effect and a random intercept for each subject shows that the two untrustworthy proposers as well as the male neutral proposer elicit a higher discounting rate for the uncertain option (see Table 5). Hence, in these conditions the participant tends to devalue probabilistic options more steeply compared to the baseline decision style. Figure 5A reports the back-transformed model estimates of h parameters.

We then apply the second mixed effect model on the log-transformed h parameter with gender and trustworthiness of the proposer as fixed effects and a random intercept for each subject. Again, we found that the level of trustworthiness is the only significant effect of the model (Table 6). A post hoc comparison confirmed that untrustworthy proposers elicit a higher h parameter (4.29 ± 0.48) compared to neutral (3.26 ± 0.36) and trustworthy proposers (2.70 ± 0.30), but also that trustworthy proposers elicit a significantly lower h compared to neutral proposers.

#### 3.2.2. Response Times

In this task, the average response time is 2.42 (SD = 1.51) seconds, 2.82 (1.61) seconds without a face, and 2.35 (1.49) seconds with a face. A generalized mixed effect model with family set to inverse Gaussian and identity link function is used to test the effect of the presence of each proposer against the baseline condition on participants’ response times, leaving a random intercept for each subject. Similar to what we found for the previous task, all coefficients are significant and negative, meaning that response times are faster when a proposer is shown with the choice (see Table 7).

**Figure 6:**
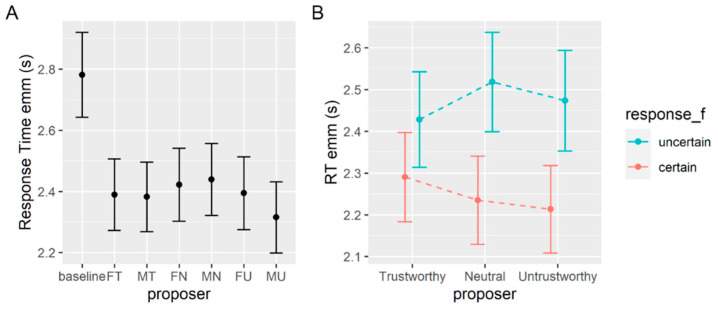
(**A**) Estimated marginal means of response times in seconds for the baseline and each experimental condition in the probabilistic discounting task. (**B**) Estimated marginal means of response times for each level of trustworthiness when the preference was for the certain (red) or uncertain (blue) options.

A second generalized mixed effect model is used to test for the different contributions of the factors at play. We model the response times with proposer trustworthiness and gender and with the given response (preference for the certain or uncertain alternative) as fixed factors, and with a random intercept for each subject. An omnibus Wald chi square test shows a significant main effect of a given response and a significant two-way interaction between response and level of trustworthiness (see Table 8). Post hoc comparisons show that response times are higher when participants prefer the uncertain option (2.47 ± 0.05) compared to the certain option (2.25 ± 0.05). In particular, the difference is bigger when the proposer is either neutral (mean difference = 0.28, *p* < 0.001) and untrustworthy (mean difference = 0.26, *p* < 0.001), rather than trustworthy (mean difference = 0.14, *p* < 0.001). See Figure 6A for model estimates of each condition.

### 3.3. Oxytocin Receptor DNA Methylation and Discounting Behavior

Firstly, for each participant, two delta scores are computed by subtracting the mean k (or the mean h) obtained in the two untrustworthy conditions to the k value (or the h value) obtained in the baseline condition, as reported in Formula (3).

Δ_k (h)_ = k (or h) *_untrustworthy_* − k (or h) *_baseline_*, (3)

Thus, a delta score near to 0 indicates no differences between conditions, a delta score above 0 indicates that the participant discounted more when there was an untrustworthy proposer, and a delta score below 0 indicates that the participant discounted less when there was an untrustworthy proposer. As a consequence, the more positive the delta score the more the untrustworthy proposer presence led the participant to prefer the immediate reward over the delayed one and to prefer the sure reward over the not sure one.

The obtained delta scores are then used to calculate a Pearson’s product moment correlation coefficient between those scores and the level of *OXTR* DNA methylation in four different CpG sites (site 1, 2, 3, and 4). Results show a statistically significant positive correlation between the percentage of methylation on site 3 and the Δ_k_ score (r = 0.361, *n* = 51, *p* = 0.005), suggesting that the greater the level of methylation, the stronger the effect of the presence of an untrustworthy proposer on the participant’s delay discounting.

To test the hypothesis that the participants’ methylation level on site 3 predicts the temporal discounting at the presence of an untrustworthy proposer, and to further verify the effect of baseline discounting on this relationship, a moderation model is performed by using PROCESS [52]. Within PROCESS, we select model 1 as representative of our hypothesized model and we use a 1000 resamples bootstrap method with the confidence interval set to 95%. In the moderation model, the site 3 methylation level is entered as the predictor (X) while the k parameter in the untrustworthy condition functions as the outcome (Y). Participants’ k parameter in the baseline (low, middle, high) is added as moderator (M).

**Figure 7:**
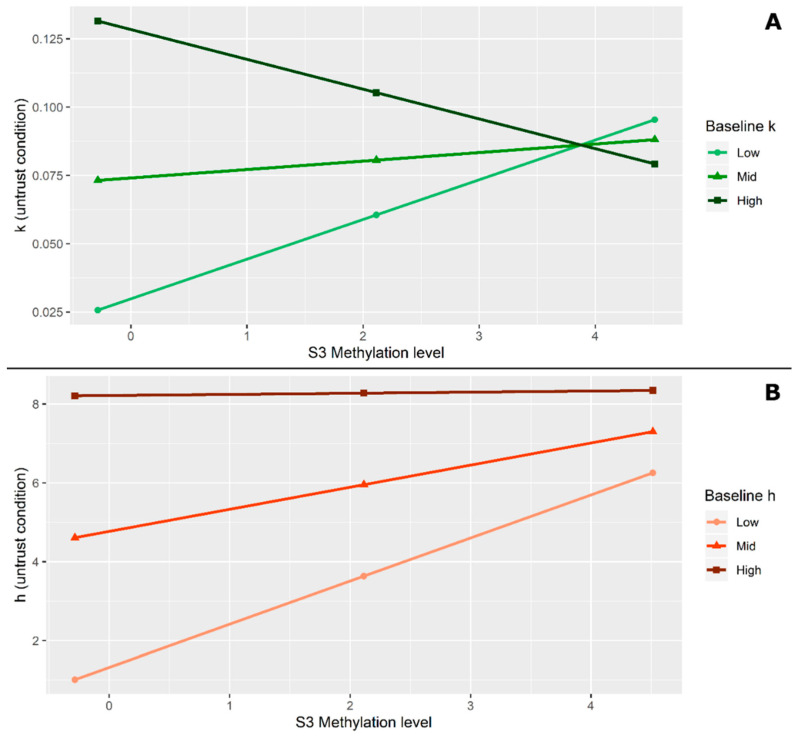
(**A**) Effect of the moderator (baseline k) on the relationship between site 3 methylation level and k in the untrustworthy condition. (**B**) Effect of the moderator (h baseline) on the relationship between site 3 methylation level and h in the untrustworthy condition.

Results show that the moderation model is significant with F_3,47_ = 4.41, *p* = 0.008, R^2^ = 0.22. Moreover, the conditional effect of X (site 3 methylation) on Y (k in untrustworthy conditions) at different values of the moderator (baseline k) reveals that the effect of the site 3 methylation level on increasing the discount rate in untrustworthy conditions is significant only for participants with lower (compared to middle and high) baseline k (with t = 2.64, *p* = 0.01; 95% CI: LLCI = 0.0035; ULCI = 0.0256) suggesting that the more participants with low baseline k show methylation on site 3, the more they discount the delayed reward when options are proposed by an untrustworthy face (see Figure 7A).

Similarly, a positive Pearson’s correlation was found between the percentage of methylation on site 3 and the Δ_h_ score (r = 0.431, *n* = 51, *p* = 0.001). In this case, results suggest that greater levels of methylation are associated with more preferences for the sure options when there is an untrustworthy proposer. To further explore this relationship a moderation model by using the site 3 methylation level as the predictor (X), the h parameter in the baseline (low, middle, high) as moderator (M), and the h parameter in the untrustworthy condition as outcome (Y) is performed.

Results show the significance for the model with F_3,47_ = 14.96, *p* = 0.0000, R^2^ = 0.488 and the significant conditional effect of X on Y at two different values of the moderator: when participants present lower baseline h (with t = 4.509, *p* = 0.000; 95% CI: LLCI = 0.6048; ULCI = 1.579) and middle baseline h (with t = 2.674, *p* = 0.01; 95% CI: LLCI = 0.1389; ULCI = 0.9817) suggesting that the higher level of site 3 methylation generally explains the higher level of probability discount in the untrustworthy condition except when considering participants who already have high h parameters in the baseline (see Figure 7B).

## 4. Discussion

We here observed that the presence of a risky factor such as untrustworthiness activates a protective behavioral pattern, represented by discounting parameters, and this shift towards protective choices is stronger in individuals with altered DNA methylation at the OXTR gene level.

The first set of analyses put the light on the role of the proposer’s trustworthiness in influencing explicit choices. As presented in the previous section, participants’ explicit choices in the baseline condition significantly differ from various conditions in which trustworthy or untrustworthy faces were introduced as proposers.

For what concerns discounting parameters, results seem to confirm our initial hypothesis that the presence of an untrustworthy proposer would produce a decrease in the availability to wait or to risk (read an increase in k and h) since participants showed more preferences for sooner smaller rewards (“Now”) and for certain smaller rewards (“Sure”) in the untrustworthy conditions. In the delay discounting task, the neutral male proposer (*p* = 0.008), the untrustworthy female proposer (*p* < 0.001), and the untrustworthy male proposer (*p* < 0.001) induced a higher discounting rate compared to the baseline condition (see Figure 3A). The same three proposers induced a higher discounting rate in the probability discounting task when compared to the baseline condition (*p* < 0.001 for all three, see Figure 5A). When looking at the effects within the “face” conditions of the tasks, we found in both a significant effect of the trustworthiness level on the discounting parameters (*p* < 0.001, see Figure 3B and Figure 5B), with untrustworthy proposers inducing higher discounting rates compared to trustworthy and neutral ones. Surprisingly, even neutral proposers seemed to produce an increase in the h discounting parameter when compared to the trustworthy proposers, but this is only significant for the probability discounting task. A similarly surprising result was a significant effect of the gender of the proposer on the discounting rate for the delay discounting task (*p* = 0.013). These results suggest that the bare presence of the proposer’s face induces changes in the availability to wait and risk, so more preferences are for immediate or certain rewards, and that this effect is stronger when the proposer is perceived as untrustworthy. It is not clear how to interpretate the effects observed based on the gender of the proposer, since we have found these just for the temporal discounting parameter, and we do not observe it in the response times.

Thus, results coming from the explicit data analysis of both tasks suggest a difference in the way the presence of a proposer’s face, particularly an untrustworthy one, affects both temporal and probabilistic discounting. Indeed, from a temporal point of view, an untrustworthy proposer promotes a protective choice pattern directing choices toward the immediate option to ensure at least a small reward; from a probabilistic point of view, an untrustworthy proposer promotes a protective choice pattern by directing preferences toward sure options.

The second set of analyses aimed to verify whether implicit response times reflected choice patterns between conditions.

In both tasks, all the choices made when the face of a proposer was shown were faster when compared to the baseline condition (all *p* < 0.001, except for MT, MN, and FN in the delay discounting task where *p* < 0.01, see Figure 4A and Figure 6A). As a possible explanation, it could be that the proposer’s face is a salient source of information that makes the decision-making process easier and thus faster. A similar facilitation effect given by the social nature of the stimuli used is well described in the Wason selection task, which is often difficult to correctly solve in its numerical form, and is instead easily solved when put in social terms [54].

When we look at the differences within the experimental conditions (with proposers), the main result that we find is a difference in the time it takes to go for the immediate or certain option compared to the delayed (*p* < 0.001, Figure 4B) or uncertain one (*p* < 0.001, Figure 6B). In the probabilistic discounting task, we also observe a small interaction effect between the given response and the proposer’s level of trustworthiness in that trial. When participants prefer the uncertain option, the level of trustworthiness does not make much difference in the time required to decide. By contrast, they appear to be quicker to go for the certain answer when the proposer is untrustworthy compared to trustworthy. Analyzing the temporal discounting task, although we do not find a significant interaction effect, the trend in response times appears to be similar to what we find in the probabilistic task.

Finally, the hypothesis that the OXTR gene methylation level would explain differences in the effect of an untrustworthy proposer on decision-making has been tested. The final sample size at this stage of the analysis was 48, due to technical problems with the salivary sample collection. Despite the limited number of samples, both models reported a good level of explained variance, especially when comparing it with previous works [55], thus motivating further future investigation with lager samples. The results seem to confirm a strong role of one of the CpG sites analyzed in the increase in k and h produced by an untrustworthy proposer, particularly for participants who presented a lower baseline k and for participants who presented a lower and middle baseline h.

Thus, our results seem to suggest that the higher the level of DNA methylation at the *OXTR* gene, the higher the influence of the proposer’s social features (i.e., trustworthiness) in the decision-making process.

These promising results are partially confirmed by some evidence on similar topics. For example, it was recently shown how a higher *OXTR* methylation level is linked to enhanced infants’ brain responses (as measured via fNIRS) to angry and fearful faces [55], while other evidence suggests that higher *OXTR* methylation is associated with problems with facial and emotional recognition [56]. While there is some evidence that the role of oxytocin administration in social discounting may be modulated by empathy traits [57], to the best of our knowledge there is only one paper directly investigating the role of oxytocin administration in reducing temporal discounting in a sample affected by social anxiety disorder [58].

In the wider scenario, it is difficult to draw conclusions on the role of OXTR in social and emotional processing given the strong heterogeneity of the used experimental designs, samples, and measures, as well as the different *OXTR* foci when in (epi)genetic settings [59].

Evidence coming from our study helps to highlight that current models of discounting behavior (such as impulsivity-based ones) should consider more social variables in pondering and selecting an option during decision-making. This is particularly true when decisions are based on others’ behaviors (e.g., the proposer will or will not deliver the promised delayed option in the future), since it is more likely that these will be predicted from interpersonal representations (such as the proposer’s perceived trustworthiness). This could be a particularly crucial point to be tested in future experiments. Even independently from the epigenetics results, we believe our behavioral results contribute to the theorical debate on variables affecting discounting behavior, suggesting that future studies should produce more data on the interaction between individual baseline tendencies in discounting (i.e., more or less discounting) and social variables. Hence, investigating the role of this interaction in keeping stable or not discounting parameters over time would be fundamental in clinical applications.

Finally, out of clinical applications, future studies should better investigate the role of the proposer’s features on discounting choice patterns as related to real-life decisions (e.g., decisions related to health, education, nutrition, savings, etc.).

## 5. Conclusions

We here studied whether the presence of proposers and their level of perceived trustworthiness could influence discounting behavior compared to a baseline, and if OXTR gene DNA methylation levels could related to these effects.

Behavioral results show that the presence of untrustworthy proposers significantly increases participants’ discounting rates, meaning that they are less prone to wait or to risk for a larger reward, and the decision to go for the immediate/certain option is associated to faster response times in both tasks.

We also show with a moderation analysis that higher levels of OXTR gene methylation are linked to a higher impact of proposer’s social features such as perceived untrustworthiness, especially for participants who started with lower baseline levels.

Although limited in sample size, our study showed promising results and suggests that further investigations in social and epigenetic variables are justified and desirable.

## Figures and Tables

**Table 1:** Results of lmm on k parameter (log scale). Each coefficient is a contrast between the baseline and the condition. SE is the same for each level of proposer because the data are balanced. CI = 95%. LL = lower level. UP = upper level. Significant statistics (t as z criterion) are reported in bold.

				CI	
Term	Estimate	SE	Statistic	LL	UL
(Intercept)	−3.768	0.154	−24.472	−4.070	−3.466
Proposer: Female Trustworthy	−0.127	0.139	−0.913	−0.400	0.146
Proposer: Male Trustworthy	−0.051	0.139	−0.365	−0.324	0.222
Proposer: Female Neutral	0.094	0.139	0.675	−0.179	0.367
Proposer: Male Neutral	0.333	0.139	**2.388**	0.060	0.606
Proposer: Female Untrustworthy	0.599	0.139	**4.300**	0.326	0.872
Proposer: Male Untrustworthy	0.727	0.139	**5.218**	0.454	1.000

**Table 2:** Results of Wald χ² test for the lmm on the k parameter. df = degree of freedom. Significant *p* values are reported in bold.

	χ²	df	*p*
(Intercept)	730.18	1	**<0.001**
Trustworthiness	24.62	2	**<0.001**
Gender	6.19	1	**0.013**
Trustworthiness: Gender	1.96	2	0.375

**Table 3:** Coefficient of glmm on response times. Each coefficient is a contrast between the baseline and the condition. CI = 95%. LL = lower level. UP = upper level. Significant statistics (*t* as *z* criterion) are reported in bold.

				CI	
Term	Estimate	SE	Statistic	LL	UL
(Intercept)	3.336	0.079	**42.014**	3.180	3.492
Female Trustworthy	−0.378	0.087	**−4.329**	−0.549	−0.207
Male Trustworthy	−0.253	0.094	**−2.690**	−0.438	−0.069
Female Neutral	−0.217	0.093	**−2.336**	−0.399	−0.035
Male Neutral	−0.208	0.090	**−2.322**	−0.384	−0.032
Female Untrustworthy	−0.341	0.089	**−3.841**	−0.515	−0.167
Male Untrustworthy	−0.385	0.092	**−4.200**	−0.564	−0.205

**Table 4:** Results of Wald χ² test for the glmm on response times. df = degree of freedom. Significant *p* values are reported in bold.

	χ²	df	*p*
(Intercept)	4133.68	1	**<0.001**
Gender	3.10	1	0.078
Trustworthiness	4.53	2	0.104
Response	101.92	1	**<0.001**
Gender: Trustworthiness	2.11	2	0.349
Gender: Response	0.23	1	0.631
Trustworthiness: Response	3.85	2	0.146
Gender: Trustworthiness: Response	2.85	2	0.241

**Table 5:** Results of lmm on h parameter (log scale). SE is the same for each level of proposer because the data are balanced. CI = 95%. LL = lower level. UP = upper level. Significant statistics (t as z criterion) are reported in bold.

				CI	
Term	Estimate	SE	Statistic	LL	UL
(Intercept)	0.933	0.117	7.942	0.703	1.163
Female Trustworthy	0.021	0.091	0.236	−0.156	0.199
Male Trustworthy	0.096	0.091	1.058	−0.082	0.273
Female Neutral	0.159	0.091	1.753	−0.019	0.336
Male Neutral	0.336	0.091	**3.706**	0.158	0.513
Female Untrustworthy	0.534	0.091	**5.902**	0.357	0.712
Male Untrustworthy	0.513	0.091	**5.667**	0.336	0.691

**Table 6:** Results of Wald χ² test for the lmm on h parameters. df = degree of freedom. Significant *p* values are reported in bold.

	χ²	df	*p*
(Intercept)	132.28	1	**<0.001**
Gender	2.14	1	0.144
Trustworthiness	53.19	2	**<0.001**
Gender: Trustworthiness	2.38	2	0.304

**Table 7:** Coefficient of glmm on response times. Each coefficient is a contrast between the baseline and the condition. CI = 95%. LL = lower level. UP = upper level. Significant statistics (*t* as *z* criterion) are reported in bold.

				CI	
Term	Estimate	SE	Statistic	LL	UL
(Intercept)	2.782	0.071	**39.349**	2.643	2.920
Female Trustworthy	−0.393	0.076	**−5.190**	−0.541	−0.244
Male Trustworthy	−0.400	0.076	**−5.252**	−0.549	−0.250
Female Neutral	−0.360	0.075	**−4.794**	−0.507	−0.213
Male Neutral	−0.342	0.076	**−4.485**	−0.492	−0.193
Female Untrustworthy	−0.387	0.072	**−5.394**	−0.528	−0.247
Male Untrustworthy	−0.467	0.075	**−6.216**	−0.614	−0.320

**Table 8:** Results of Wald χ² test for the glmm on response times. df = degree of freedom. Significant *p* values are reported in bold.

	χ²	df	*p*
(Intercept)	2112.60	1	**<0.001**
Gender	0.45	1	0.503
Trustworthiness	1.42	2	0.492
Response	90.71	1	**<0.001**
Gender: Trustworthiness	0.31	2	0.857
Gender: Response	0.52	1	0.470
Trustworthiness: Response	8.56	2	**0.014**
Gender: Trustworthiness: Response	2.42	2	0.299

## Data Availability

Data and code for the analyses available on OSF at https://osf.io/rp2dc/?view_only=13fe736c6f864ab791e9e4eea5973eda (accessed on 20 November 2021).
